# Iron Status and Short-Term Recovery after Non-Severe Acute Myocarditis: A Prospective Observational Study

**DOI:** 10.3390/biomedicines11082136

**Published:** 2023-07-28

**Authors:** Paweł Franczuk, Michał Tkaczyszyn, Aneta Kosiorek, Katarzyna Kulej-Łyko, Kamil Aleksander Kobak, Monika Kasztura, Alicja Sołtowska, Joanna Jaroch, Piotr Ponikowski, Ewa Anita Jankowska

**Affiliations:** 1 Institute of Heart Diseases, Wroclaw Medical University, 50-556 Wroclaw, Poland; 2 Institute of Heart Diseases, University Hospital, 50-556 Wroclaw, Poland; 3 Aging and Metabolism Research Program, Oklahoma Medical Research Foundation, Oklahoma City, OK 73104, USA; 4 Department of Food Hygiene and Consumer Health Protection, Faculty of Veterinary Medicine, Wroclaw University of Environmental and Life Sciences, 50-375 Wroclaw, Poland; 5 Department of Cardiology, Tadeusz Marciniak Lower Silesia Specialist Hospital-Emergency Medicine Center, 54-049 Wroclaw, Poland; 6 Division of Internal Medicine Nursing, Faculty of Health Science, Wroclaw Medical University, 51-618 Wroclaw, Poland

**Keywords:** myocarditis, iron status, ferritin, hepcidin, inflammation, myocardial dysfunction

## Abstract

Pathomechanisms responsible for recovery from acute myocarditis (MCD) or progression to non-ischemic cardiomyopathy have not been comprehensively investigated. Iron, positioned at the crossroads of inflammation and the energy metabolism of cardiomyocytes, may contribute to the pathophysiology of inflammatory myocardial disease. The aim of this study was to evaluate whether systemic iron parameters are related to myocardial dysfunction in MCD patients. We prospectively enrolled 42 consecutive patients hospitalized for MCD. Their iron status and their clinical, laboratory, and echocardiographic indices were assessed during hospitalization and during ambulatory visits six weeks after discharge. A control group comprising healthy volunteers was recruited. The MCD patients had higher serum ferritin and hepcidin and lower serum iron concentration and transferrin saturation (TSAT) than the healthy controls (all *p* < 0.01). Six weeks after discharge, the iron status of the MCD patients was already comparable to that of the control group. During hospitalization, lower serum iron and TSAT correlated with higher NT-proBNP (both *p* < 0.05). In-hospital lower serum iron and TSAT correlated with both a lower left ventricular ejection fraction (LVEF) and worse left ventricular global longitudinal strain at follow-up visits (all *p* < 0.05). In conclusion, in patients with acute MCD, iron status is altered and normalizes within six weeks. Low serum iron and TSAT are related to greater in-hospital neurohormonal activation and subtle persistent left ventricular dysfunction.

## 1. Introduction

In the majority of patients with acute myocarditis (MCD), the disease spontaneously regresses without significant clinical sequelae [1,2,3]. However, there are subjects who develop post-myocarditis non-ischemic cardiomyopathy, and such patients—who are at higher risk of poor outcomes—are difficult to identify [1,2,3]. Our knowledge of the mechanisms behind recovery from MCD or the development of subsequent cardiomyopathy is limited, and various factors responsible for this process have been postulated, including innate immune competence, comorbidities, and genetics [1,4,5,6,7].

There are pathophysiological links between disordered iron status and cardiomyopathy. Both iron deficiency and iron overload are detrimental for cardiomyocytes. This provides an insight into the U-shaped relationship between a (sub-)cellular iron level and cardiomyocyte homeostasis [8,9,10,11,12,13,14,15]. An abnormal immune response is considered to be the major pathophysiological mechanism promoting MCD, and circulating biomarkers of iron metabolism are also linked to immunological reactions [1,2,16,17,18,19]. Ferritin and hepcidin are widely recognized as acute phase reactants and biomarkers of inflammation [20,21,22,23,24]. The release of inflammatory cytokines upregulates hepcidin synthesis and the secretion of iron-poor ferritin by macrophages, which eventually leads to limiting the availability of iron to microorganisms during infection [25,26,27]. Nevertheless, whether or not peripheral blood iron status relates to the recovery from acute MCD remains unexplored.

The aim of the current study was to evaluate whether systemic iron parameters are related to myocardial dysfunction in acute MCD patients.

## 2. Materials and Methods

### 2.1. Patients Population

The prospective MCD registry consisted of patients who were hospitalized consecutively for acute MCD during 2014–2019 in two tertiary referral cardiology centers: the Cardiology Department of the Center for Heart Diseases in the 4th Military Hospital in Wroclaw and the Department of Cardiology of the Tadeusz Marciniak Lower Silesia Specialist Hospital—Emergency Medicine Center in Wroclaw.

MCD was diagnosed according to the criteria presented in Table 1. The follow-up period for analysis was 6 weeks after discharge. A control group comprising healthy adult age-matched and gender-matched volunteers was recruited among hospital personnel and relatives in order to constitute a reference for laboratory tests and imaging.

The study protocol was approved by the local ethics committee (the Bioethics Committee, Wroclaw Medical University, Wroclaw, Poland). All patients provided written informed consent to participate in the study. The study was conducted in accordance with the Declaration of Helsinki.

### 2.2. Study Scheme

**Figure 1:**
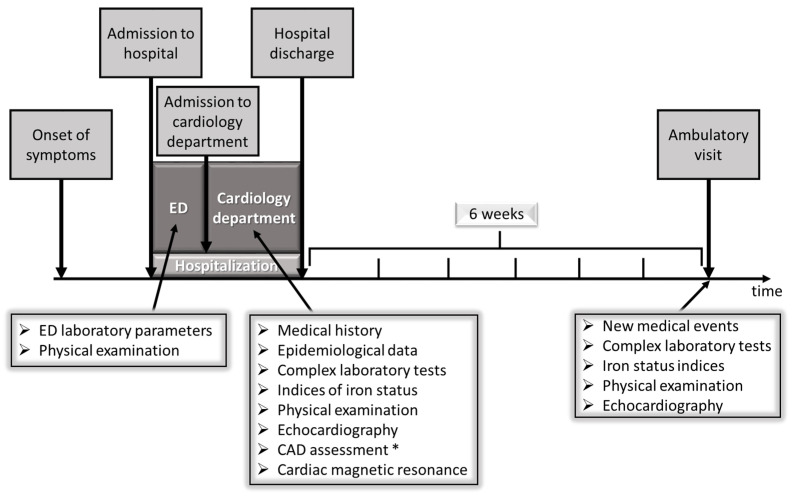
The study scheme comprised two assessments of patients with acute myocarditis during hospitalization and one at ambulatory visit after six weeks of recovery. Abbreviations: CAD, coronary artery disease; ED, Emergency Department. * coronary angiography or coronary computed tomographic angiography.

During their hospitalization for acute MCD, the patients were assessed at the emergency department and 3 ± 1 days after admission to the cardiology department. A control ambulatory visit was scheduled 6 ± 1 weeks after discharge. The study design scheme is presented in Figure 1.

### 2.3. Basic Clinical Evaluation

The demographic and anthropometric characteristics, medical history, comorbidities, and the characteristics of clinical presentation (signs, symptoms) of acute MCD were collected at the cardiology department (detailed medical history). At each timepoint (at the emergency department, at the cardiology department, and at the ambulatory visit), assessment of the vital signs and physical examination were performed. Mean systolic blood pressure, mean diastolic blood pressure, and mean heart rate were calculated as an average from 3 consecutive measurements.

### 2.4. Laboratory Parameters

We analyzed the following emergency department laboratory tests: serum hemoglobin, white blood cell count, high-sensitivity cardiac troponin I (hs-cTnI), N-terminal pro-B-type natriuretic peptide (NT-proBNP), C-reactive protein (CRP), serum creatinine, and alanine transaminase (ALT). The following basic laboratory parameters were measured - in fresh venous blood: in patients with acute MCD at the cardiology department and during ambulatory visit: serum hemoglobin, red blood/white blood cell/neutrophil/lymphocyte/monocyte count, reticulocyte hemoglobin content, reticulocyte count, hs-cTnI, NT-proBNP, CRP, ALT, serum creatinine, serum insulin, and thyroid-stimulating hormone.

The following blood biomarkers/parameters, reflecting iron metabolism, were measured directly (from fresh venous blood): serum ferritin (μg/L)—with an immunoassay based on electrochemiluminescence with the Elecsys 2010 System (Roche Diagnostics GmbH, Mannheim, Germany); iron (μg/dL); and unsaturated iron-binding capacity - based on a substrate method with Feren S (Thermo Fisher Scientific, Waltham, MA, USA). The total iron-binding capacity (μg/dL) was automatically calculated using serum iron and unsaturated iron-binding capacity. Transferrin saturation (TSAT) was calculated as the ratio of serum iron (μg/dL) and total iron-binding capacity (μg/dL), and expressed as a percentage. Soluble Transferrin Receptor (sTfR, mg/L) and hepcidin (ng/mL) were measured in frozen sera, after obtaining and preparing biological material from all study enrollees. Clotted blood samples were centrifuged and the supernatants were collected and frozen in −80 °C for further analysis. STfR was measured using immunonephelometry (Siemens Healthcare Diagnostics, Inc., Deerfield, IL, USA). Hepcidin was assessed using a commercially available, enzyme-linked immunosorbent assay (BACHEM s-1337 (Bachem AG, Bubendorf, Switzerland) for detecting human hepcidin-25, dedicated for research use only. This is a unique hepcidin ELISA kit which has been validated with a gold standard for hepcidin assessment—liquid chromatography mass spectrometry (LC MS) [28]. For accurate hepcidin detection, all samples were 5 times diluted and were within the linear range of the curve. The optical density of the samples was measured at 450 nm, with a reading time of 1 sec, using a microtiter plate reader Biotek Synergy HTX (Agilent Technologies, Inc., Santa Clara, CA, USA). For the assessment of the neurohormonal activation, plasma level of NT-proBNP (pg/mL) was measured using an immunoassay based on chemiluminescence with Dimension RxL system (Siemens Healthcare Diagnostics, Inc., Deerfield, IL, USA). For the evaluation of the inflammatory response, serum level of CRP (mg/L) was assessed using immunonephelometry with BN II System (Siemens Healthcare Diagnostics, Inc., Deerfield, IL, USA). For the assessment of the cardiomyocyte necrosis, hs-cTnI (µg/L) was measured using chemiluminescence (technology LOCI) on the Dimension EXL System (Siemens Healthcare Diagnostics, Inc., Deerfield, IL, USA). The serum hemoglobin concentration (g/dL), red or white cell indices and reticulocytes, were measured using the ADVIA 120 hematology system (Siemens Healthcare Diagnostics, Inc., Deerfield, IL, USA). Except for hepcidin, all diagnostic laboratory assessments were performed in the central laboratory of the Military Hospital in Wroclaw (Poland).

### 2.5. Echocardiography

A standard transthoracic echocardiography was performed at the cardiology department and during ambulatory visit six weeks after discharge. For the purposes of this study, the following variables were analyzed: left ventricular ejection fraction (LVEF), estimated using Simpson’s planimetric method; left ventricular global longitudinal strain (LV GLS), measured by the speckle tracking technique; indices of left ventricular diastolic function (including E/A, e’ sep, e’ lat and E/e’), assessed with the use of continuous wave and tissue doppler imaging; tricuspid annular plane systolic excursion (TAPSE) and segmental myocardial contractility dysfunction, estimated visually by an experienced sonographer.

### 2.6. Other Diagnostic Procedures

We analyzed the following other diagnostic procedures, performed during the index hospitalization in the cardiology ward:

− coronary angiography or coronary computed tomographic angiography—to exclude obstructive coronary artery disease;
− cardiac magnetic resonance—in search for the indices of MCD, to confirm the diagnosis.

### 2.7. Statistical Analyses

Most of the continuous variables had a normal distribution, and were expressed as mean (standard error of the mean). NT-proBNP, hs-cTnI, CRP, serum hepcidin, reticulocyte count, ALT, alcohol consumption, and neutrophil-to-lymphocyte ratio had a skewed distribution and were expressed as median with lower and upper quartiles (interquartile range). These variables were log-transformed (a natural logarithm, ln) before inclusion in further analyses. The intergroup differences between the patients with acute MCD and the healthy controls were tested using the *t*-test for unpaired samples. The categorical variables were expressed as numbers (with percentages). The intergroup differences regarding the categorized variables were tested using the Pearson’s Chi-square test. The associations between the iron status indices and the laboratory or echocardiographic parameters were tested using Pearson’s correlation coefficients. The changes in laboratory parameters between the hospitalization and the ambulatory visit were tested using t-test for paired samples. A value of *p* < 0.05 was considered statistically significant. All statistical analyses were performed using the Statistica 13 data analysis software system (TIBCO Software Inc., Palo Alto, CA, USA).

## 3. Results

### 3.1. Patients Characteristics

Out of 63 patients admitted to the hospital with suspected acute MCD, 21 initially evaluated subjects were excluded:

− 3 with normal level of high sensitivity cardiac troponin I;
− 18 without indices of MCD in cardiac magnetic resonance.

The final study group of patients hospitalized for acute MCD comprised 42 subjects.

The baseline demographic, clinical, laboratory, and echocardiographic characteristics of the study group versus controls, who were 15 age-matched and gender-matched healthy volunteers, are presented in Table 2. Co-morbidities were not prevalent in the patients with acute MCD. Only three (7%) of them had a history of a previous MCD. During the hospitalization and the subsequent observation, none of the subjects died, was rehospitalized for unplanned reasons (including cardiovascular and non-cardiovascular causes), nor required a heart transplantation or a ventricular assist device.

### 3.2. Clinical Manifestation of MCD

The most prevalent symptoms of acute MCD were chest pain—in 39 (93%) patients, dyspnea—in 13 (31%), and palpitations—in 6 (14%). Two (5%) patients presented symptoms of acute heart failure at the time of admission. Within 30 days before admission to the hospital, fever (T > 38 °C) was present in 26 (62%) of the patients, pharyngitis in 25 (60%), arthritis in 22 (52%), cough in 18 (43%), rhinitis in 15 (36%), nausea/vomiting in 10 (24%), stomach pain in 4 (10%), diarrhea in 3 (7%), and sinusitis, rash, and dysuria in two (5%) patients. Twenty-two (52%) subjects still presented symptoms of infection at the time of admission.

In transthoracic echocardiography, the patients hospitalized for acute MCD presented lower LVEF, higher LV GLS, and worse parameters of diastolic function—lower values of E/A, e’ lat and higher of E/e’—compared to the control group (Table 2). A segmental myocardial contractility dysfunction was present in more than half of the subjects.

### 3.3. Follow-Up Visit

The ambulatory visit at six weeks after discharge was missed by 7 patients—data for 35 subjects were available. No differences regarding baseline clinical characteristics were found between the 35 patients, who attended the ambulatory visit, and the 7 patients, who were lost to follow-up.

**Figure 2:**
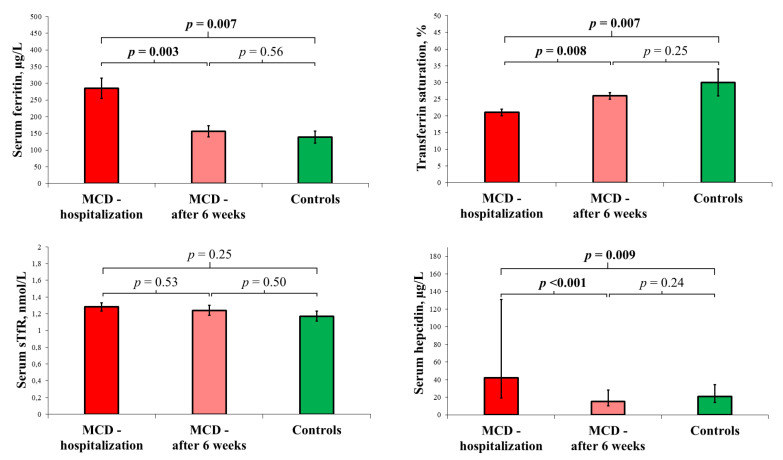
Serum ferritin, transferrin saturation, serum soluble transferrin receptor (mean (bar) with SEM (whiskers)), and serum hepcidin (median (bar) with interquartile range (whiskers)) in the patients with acute myocarditis during hospitalization (*n* = 42) and at the follow-up visit 6 weeks after discharge (*n* = 35), in comparison with age- and gender-matched healthy volunteers (*n* = 15). The MCD patients during hospitalization had higher serum ferritin and hepcidin and lower transferrin saturation than the healthy controls. Six weeks after discharge, in the MCD patients, serum ferritin and hepcidin decreased, while transferrin saturation increased, and the iron status was already comparable to that of the control group. For details, see “Section 2.7”.

At the ambulatory visit, NT-proBNP, high-sensitivity troponin I, and CRP were lower than during the hospitalization (all *p*-values < 0.001), and already comparable to the healthy controls (*p*-values: 0.36, 0.16, 0.17, respectively). The changes in the indices of iron status after six weeks of recovery together with the comparison to the control group are presented in Figure 2.

### 3.4. Relationship of Neurohormonal Activation, Cardiomyocyte Necrosis, Inflammatory Response, and Cardiac Dysfunction with In-Hospital Iron Status

The relationships of the in-hospital indices of iron status with the in-hospital laboratory markers of neurohormonal activation, cardiomyocyte necrosis, and inflammatory response, and with the echocardiographic measures of cardiac dysfunction from the index hospitalization, are presented in Table 3. 

During the index hospitalization, serum iron and acute phase reactants (ferritin and hepcidin) correlated with CRP. Lower in-hospital serum iron and TSAT were associated with higher in-hospital NT-proBNP.

Moreover, an analysis of echocardiographic evaluation, performed six weeks after discharge, demonstrated that lower serum iron and TSAT, measured during the hospitalization, were associated with a persistent left ventricular dysfunction (lower LVEF and higher LV GLS) at the follow-up visit (Table 4).

The levels of hs-TnI, NT-proBNP, LVEF and LV GLS, assessed during the follow-up visit, were neither related to CRP, nor to white blood cell indices (white blood cell count, neutrophil, lymphocyte or monocyte count and neutrophil-to-lymphocyte ratio) from the index hospitalization.

## 4. Discussion

The major findings arising from the current study are (1) the patients with acute MCD had comparable red blood cell indices but altered indices of iron status, compared with the healthy controls, (2) disordered iron parameters normalized within six weeks after the index hospitalization, (3) low serum iron and TSAT, which constitute indices of iron deficiency but not acute phase reactants, were correlated with greater in-hospital neurohormonal activation (measured via the concentration of natriuretic peptides), together with a subtle persistent left ventricular dysfunction, detected after six weeks of recovery (assessed via LVEF and LV GLS).

There is evidence, from experimental animal and cell cultures, that systemic iron deficiency relates to multifaceted myocardial dysfunction [8,10,12,18]. For example, systemic ID, induced in animals through bleeding or an iron-depleted diet, resulted in mitochondrial dysfunction, abnormal intracellular metabolism, and destruction or apoptosis of cardiomyocytes [29,30,31,32]. All those adverse alterations were associated with progression to cardiomyopathy with systolic dysfunction, and development of overt heart failure [12,33,34]. Recently, experimental models have demonstrated that the detrimental impact of iron deficiency on cardiac muscle can be reversed via iron supplementation [11,35,36]. In addition, clinical data have confirmed beneficial effects of iron supplementation in iron-deficient subjects in chronic conditions [37,38]. In patients with heart failure with left ventricular dysfunction, intravenous iron therapy leaded to improvement of symptoms, exercise capacity, and quality of life in randomized clinical trials, and was even associated with better prognosis in further meta-analysis [37,38]. Recently, clinical data have shown favorable results of treatment of iron depletion in a post-acute clinical scenario: in patients who were stabilized after an episode of acute heart failure, a treatment with ferric carboxymaltose reduced the risk of heart failure hospitalizations in a randomized clinical trial [39]. Whether iron supplementation in patients with acute MCD, with peripheral indices of iron deficiency, may be beneficial and prevent progression to post-myocarditis non-ischemic cardiomyopathy, has never been explored. It may be an interesting direction for future research.

Although we have no data on the cardiomyocyte iron status in patients with acute MCD, there have been documented links between the systemic iron status and myocardial iron metabolism [31,40,41]. Cardiomyocytes cultured in iron-depleted environment were revealed to exhibit an overexpression of transferrin receptor 1, which constitutes evidence of an increased intracellular iron demand [31]. What is more, the overexpressed transferrin receptor 1 was related to an increased apoptosis of cardiomyocytes [31,32]. In human cardiomyocytes, a decreased iron content impairs the mitochondrial respiration, leading to both contractile and relaxation dysfunction [11]. Myocardial iron deficiency is closely linked to left ventricular dilation and fibrosis, resulting in cardiomyopathy and heart failure [12,40].

Furthermore, relationships between iron deficiency and an impairment of antioxidative defense have been postulated [42]. Iron constitutes a metal cofactor for mitochondrial enzymes, responsible for antioxidative processes. Several studies have demonstrated the development of mitochondrial dysfunction under iron-depleted conditions [11,42]. Moreover, the most vulnerable to iron restriction are the cells of high mitogenic potential (i.e., immune cells) or of high energy demand (i.e., cardiomyocytes) [16]. Hence, there are premises to consider iron metabolism to be an important contributor to the complex pathophysiology of MCD.

Iron metabolism plays an important role in the pathogenesis of inflammation [26]. Both ferritin and hepcidin are known positive acute-phase proteins, which means that their plasma concentrations increase during an inflammatory process, and our analyses have confirmed high serum hepcidin and ferritin levels in the patients with acute MCD [20,21,22,25]. The aim of the alterations in iron status during the inflammation is to limit iron availability to microorganisms in the course of infection. The crucial mechanism, involving iron metabolism into inflammation, seems to be the upregulation of hepcidin, a hormone synthesized predominantly by hepatocytes, by released inflammatory cytokines [26,27]. The circulating hepcidin, via a specific receptor (ferroportin), downregulates the expression of proteins responsible for the import of iron into enterocytes and, by internalizing ferroportin, also disables further export of the intracellular iron to the circulating transferrin [25,26,27]. As a consequence, it decreases the iron absorption by enterocytes, and additionally reduces the export of the intracellular iron, trapped mainly in hepatocytes and macrophages as iron-rich ferritin, leading to a reduction in the concentration of serum iron [26,27]. On the other hand, iron-poor ferritin is secreted by macrophages into circulation, resulting in high serum ferritin levels [26]. Although the abundant store of intracellular iron results in downregulation of transferrin production and in lowering total iron binding capacity, the extensive decrease in serum iron prevails over this mechanism, leading to a lowering of TSAT [26,27]. 

Our study has demonstrated an association of lower serum iron and TSAT with greater in-hospital neurohormonal activation and with a subtle deterioration of left ventricular function after six weeks of observation. Considering the aforementioned mechanisms of the pathophysiology of inflammation, our finding may be explained by the fact that the decrease in TSAT and in serum iron concentration may reflect an enhanced myocardial inflammation and a more extensive affection of the myocardium. Furthermore, functional iron deficiency, expressed by low TSAT, can derange myocardial energetics. In consequence, it may adversely affect the processes of healing the damaged myocardial tissue, and thus impair recovery from acute MCD. What is of note, CRP—a biomarker of inflammation routinely used in clinical practice—was related neither to the in-hospital neurohormonal activation nor to the subtle persistent left ventricular dysfunction. The reason for the difference between CRP and TSAT, or serum iron concentration, may be the fact that, in contrast to CRP, the analyzed iron parameters express cumulatively both the intensity of the inflammation and the disturbed myocardial energetics, and thus, they better manifest the defected recovery process. These indices of iron status may reflect multiple negative mechanisms during the recovery, not only those concerning the inflammatory response.

Further studies and longer periods of observation are required to determine the relationships between the iron metabolism during acute MCD and the risk of an unfavorable progression to post-myocarditis non-ischemic cardiomyopathy. However, the assessment of circulating biomarkers of iron status may become a valuable tool in risk stratification and the evaluation of recovery after acute MCD. Moreover, the treatment of iron deficiency in MCD patients could constitute an interesting area for the future clinical trials.

### Limitations of the Study

The current study has certain limitations, which should be punctuated. Firstly, although the recruitment was conducted in two cardiology centers, and consecutive patients hospitalized for acute MCD were enrolled, the final study group comprised a relatively small number of subjects, and thus only basic statistical analyses were performed. However, it needs to be acknowledged that the previous research into MCD has mainly comprised studies of small-scale populations, above all, due to the relatively low incidence of this disease. Therefore, extensive multicenter trials in larger populations are greatly needed. Secondly, we assessed indices of peripheral blood iron status, and we have no data on the myocardial iron status, which may be crucial for a better recognition of the role of iron in the pathobiology of myocardial inflammation. Eventually, to evaluate the inflammatory reactions, we utilized only C-reactive protein concentrations, while a complex assessment of inflammatory indices, including concentrations of pro-inflammatory interleukins, would give a superior insight into the contribution of the immune response, its relationships with iron metabolism, and its associations with recovery from acute MCD.

## 5. Conclusions

Patients hospitalized for acute MCD have comparable red blood cell indices, but altered indices of iron status, compared with healthy controls. The deranged iron status normalizes within six weeks of recovery. Lower serum iron and TSAT (indicators of iron deficiency other than acute phase reactants) correlate with a greater in-hospital neurohormonal activation and a subtle persistent left ventricular dysfunction.

## Figures and Tables

**Table 1:** The diagnostic criteria for acute myocarditis in the current study.

All Diagnostic Criteria for Acute Myocarditis Must Have Been Met:
(1) new onset symptoms suggestive of myocarditis (shortness of breath, effort intolerance, fatigue, palpitations, or chest pain),
(2) elevated high sensitivity cardiac troponin I,
(3) exclusion of obstructive coronary artery disease in coronary angiography or coronary computed tomographic angiography,
(4) features suggestive of myocarditis in cardiac magnetic resonance, and
(5) age ≥ 18 years.

**Table 2:** The baseline demographic and clinical characteristics, laboratory parameters and echocardiographic indices in patients hospitalized for acute myocarditis in comparison with a control group of age- and gender-matched healthy volunteers.

Variables, Units	MCD Patients (*n* = 42)	Control Group (*n* = 15)	*p*-Value
Age, year	32 (1)	31 (1)	0.66
Male gender, yes	41 (97%)	13 (87%)	0.10
Smoking, packet-year	0.2 (0.0–6.5)	0.0 (0.0–0.0)	0.20
Alcohol, g per week	90 (25–155)	25 (25–50)	0.01
SBP on admission, mmHg	135 (3)	118 (3)	0.003
DBP on admission, mmHg	80 (3)	77 (3)	0.62
HR on admission, mmHg	86 (3)	73 (4)	0.02
Basic laboratory parameters (cardiology department)			
Serum hemoglobin, g/dL	15.0 (0.2)	15.2 (0.3)	0.59
Red blood cell count, 10^6^/µL	5.0 (0.1)	5.0 (0.1)	0.65
White blood cell count, 10^3^/µL	7.9 (0.4)	6.3 (0.4)	0.03
Neutrophil count, 10^3^/µL	5.0 (0.3)	3.2 (0.3)	0.004
Lymphocyte count, 10^3^/µL	2.0 (0.1)	2.3 (0.2)	0.19
Monocyte count, 10^3^/µL	0.7 (0.1)	0.4 (0.1)	0.008
Neutrophil-to-lymphocyte ratio	2.3 (1.9–3.0)	1.2 (1.1–1.9)	<0.001
Reticulocyte hemoglobin content, pg	30 (3)	37 (5)	0.03
Reticulocytes, %	0.98 (0.82–1.30)	1.26 (1.06–1.56)	0.60
C-reactive protein, mg/L	54 (5)	3 (0)	<0.001
NT-proBNP, pg/mL	340 (185–594)	31 (18–46)	<0.001
Hs-cTnI, µg/L	2.44 (0.53–6.33)	0.01 (0.01–0.01)	<0.001
Serum creatinine, mg/dL	0.91 (0.02)	0.98 (0.03)	0.11
ALT, U/L	38 (27–48)	20 (13–30)	<0.001
Serum insulin, uIU/mL	13.4 (1.1)	7.9 (1.3)	0.01
TSH, mIU/L	2.0 (0.2)	2.7 (0.3)	0.07
Iron status indices			
Serum iron, µg/dL	70 (5)	101 (12)	0.006
Serum ferritin, µg/L	285 (30)	139 (18)	0.007
TSAT, %	21 (1)	30 (4)	0.007
sTfR, nmol/L	1.28 (0.05)	1.17 (0.06)	0.25
Serum hepcidin, µg/L	44 (24–131)	21 (13–44)	0.009
Transthoracic echocardiography			
LVEF, %	56 (1)	63 (1)	0.01
TAPSE, mm	21 (1)	23 (1)	0.045

The data are presented as mean (SEM) or median with interquartile range for continuous variables and counts (percentages) for nominal variables. Abbreviations: ALT, alanine transaminase; BMI, body mass index; DBP, diastolic blood pressure; HR, heart rate; hs-cTnI, high-sensitivity cardiac troponin I; LVEF, left ventricular ejection fraction; NT-proBNP, N-terminal pro B-type natriuretic peptide; SBP, systolic blood pressure; sTfR, soluble transferrin receptor; TAPSE, tricuspid annular plane systolic excursion; TSAT, transferrin saturation; TSH, thyroid-stimulating hormone. The conversion factors to SI units are as follows: for hemoglobin (g/dL), 10; for white blood cells, neutrophils, lymphocytes, and monocyte count (all in 103/µL) 1; for NT-proBNP (in pg/mL), 0.118; for serum creatinine (in mg/dL), 88.42; for serum insulin (in uIU/mL), 6.945; for serum iron (in µg/dL), 0.179; for serum ferritin (in µg/L), 2.247; for serum hepcidin (in µg/L), 0.3584.

**Table 3:** The relationships between the baseline iron indices, the biomarkers of neurohormonal activation, cardiomyocyte necrosis or inflammation, and the main laboratory or echocardiographic parameters from the index hospitalization in the patients with acute myocarditis.

	Cardiology Department					
Variables #, Units	NT-proBNP, 1 ln pg/mL	Hs-cTnI, 1 ln µg/L	CRP, 1 ln mg/L	LVEF, 1%	LV GLS, 1	TAPSE, 1 mm
Serum iron, 1 µg/dL	−0.345 *	−0.086	−0.315 *	0.169	−0.029	0.037
Serum ferritin, 1 µg/L	0.093	0.155	0.309 *	0.019	−0.135	0.167
TSAT, 1%	−0.319 *	0.018	−0.281	0.223	−0.093	0.122
sTfR, 1 nmol/L	−0.101	0.094	0.052	−0.020	0.235	−0.101
Serum hepcidin, 1 ln µg/L	0.283	0.095	0.308 *	−0.113	−0.196	0.229
NT-proBNP, 1 ln pg/mL	X	−0.022	0.166	−0.614 ***	0.111	−0.031
Hs-TnI, 1 ln µg/L	−0.022	X	0.245	0.082	0.188	0.078
CRP, 1 ln mg/L	0.166	0.245	X	−0.029	0.040	−0.173

Data are presented as Pearson’s correlation coefficient. * *p*-value < 0.05, *** *p*-value < 0.001. # Assessed at the cardiology department. Abbreviations: see Table 2; LV GLS, left ventricular global longitudinal strain. Conversion factors to SI units: see Table 2.

**Table 4:** The correlation of the baseline iron indices with the biomarkers of neurohormonal activation, cardiomyocyte necrosis or inflammation, and with echocardiographic parameters after six weeks of recovery in the patients with acute myocarditis.

Baseline	6 Weeks after Hospital Discharge					
Variables, Units	NT-proBNP, 1 ln pg/mL	hs-cTnI, 1 ln µg/L	CRP, 1 ln mg/L	LVEF, 1%	LV GLS, 1	TAPSE, 1 mm
Serum iron, 1 µg/dL	−0.012	−0.014	−0.067	0.388 *	−0.392 *	0.327
Serum ferritin, 1 µg/L	−0.104	−0.020	0.325	0.134	−0.279	0.126
TSAT, 1%	0.031	0.037	−0.104	0.391 *	−0.408 *	0.285
sTfR, 1 nmol/L	−0.013	0.219	−0.213	−0.209	0.241	0.067
Serum hepcidin, 1 ln µg/L	−0.263	−0.034	0.110	0.179	0.073	0.142

Data are presented as Pearson’s correlation coefficient. * *p*-value < 0.05. Abbreviations: see Table 2 and Table 3. Conversion factors to SI units: see Table 2.

## Data Availability

The datasets used and/or analyzed during the current study are available from the corresponding author on reasonable request.
